# miR-34a in PFIC: Hypothesized Links to Dyslipidemia and Liver Regeneration

**DOI:** 10.34172/apb.46776

**Published:** 2026-05-19

**Authors:** Amr Ahmed El-Arabey, Kapil K. Upadhyay

**Affiliations:** ^1^Pharmacology and Toxicology Department, Faculty of Pharmacy, King Khalid University, 61421, Abha, Saudi Arabia; ^2^Innovation Ecosystem Advisor, King Khalid University, 61413, Abha, Saudi Arabia; ^3^Applied College, King Khalid University, P.O. Box 9004, Abha 61413, Saudi Arabia; ^4^Division of Gastroenterology and Hepatology, Department of Internal Medicine, University of Michigan, Ann Arbor, USA

## To Editor,

 Progressive Familial Intrahepatic Cholestasis (PFIC), also known as Byler syndrome, is a rare group of autosomal recessive liver disorders characterized by early-onset, severe, and progressive cholestasis. This disease disrupts bile secretion from hepatocytes, resulting in intrahepatic accumulation of toxic bile acids, driving progressive liver injury that culminates in fibrosis, cirrhosis, and liver failure. PFIC is estimated to affect approximately 1 in 50,000 to 100,000 individuals worldwide. The molecular basis of PFIC typically involves mutations in genes encoding key bile transporters like ATP8B1, ABCB11, and ABCB4, essential for normal bile formation and export. Although defects in these genes leads to overlapping clinical and biochemical features, they also form the basis for the classification of inherited cholestatic liver diseases.^1^ Diagnosis of PFIC relies on comprehensive clinical evaluation, laboratory and genetic testing, and, increasingly, the application of non-invasive biomarkers. In recent years, Micro ribonucleic acid (miR), particularly miR-34a, have emerged as key mediators of hepatic inflammation and dysfunction, extending their relevance beyond adult cirrhosis and metabolic dysfunction-associated steatotic liver disease (MASLD) to pediatric and genetic cholestatic disorders.^2^ Growing evidence from MASLD demonstrates consistently elevated hepatic and circulating miR-34a correlating with disease severity, suggesting its dual role as a pathobiology mediator and diagnostic marker.^2^ This context heightens the significance of reports describing substantially increased serum miR-34a in PFIC patients relative to healthy controls (mean fold change around 3.39 in PFIC versus 0.6 in controls; *p value* = 0.0327).^3^ While these data collectively underscore an important association, there remains a pressing need for rigorous investigation to establish causality, clarify mechanistic pathways, and validate the practical utility of miR-34a measurement for disease monitoring and longitudinal risk stratification.^3^ Beyond its hepatic implications, PFIC possess several challenges for cardiometabolic health. Patients with PFIC experience atypical dyslipidemia, not as overt hypercholesterolemia, but rather as the paradoxical buildup of triglyceride-rich low-density lipoproteins (LDL) and suppressed high-density lipoproteins (HDL), a phenotype distinct from adult cholestatic liver disease but nonetheless capable of driving atherogenesis.^4^ Prior studies in congenital cholestasis utilized surrogate subclinical indicators, yet robust registry data and prospective trials remain limited concerning cardiovascular events and outcomes in PFIC.^5^ Critically, the molecular connection between hepatic and vascular injury may be solidified by miR-34a. Research demonstrate that miR-34a modulates macrophage cholesterol metabolism and inflammatory polarization, specifically enhancing M1 (pro-inflammatory) and restraining M2 (anti-inflammatory) macrophage phenotypes by influencing liver X receptor α (LXRα), a key regulator of cholesterol homeostasis.^6^ This pivotal role positions miR-34a not only as a modulator of hepatic inflammation but also as a key connecting link between dysregulated lipid metabolism and vascular injury.^4^ Despite these mechanistic insights, there is currently no direct clinical evidence linking circulating miR-34a levels to atherosclerotic or subclinical cardiovascular outcomes in PFIC, so its proposed cardiometabolic role should be considered a hypothesis that requires validation in well-designed prospective studies. Furthermore, the dyslipidemic profile seen in PFIC must be approached with caution, since it may differ among subtypes (ATP8B1, ABCB11, and ABCB4), and comprehensive quantitative data delineating these variations are currently limited. miR-34a plays a complex role in PFIC by affecting both liver regeneration and bile acid production. During liver regrowth after significant loss (e.g., partial hepatectomy), miR-34a expression surges, inhibiting the activin member INHBB and hepatocyte growth factor receptor MET, thereby impairing proliferation at critical junctures.^7^ Simultaneously, miR-34a has been reported to upregulate hepatic cholesterol 7α-hydroxylase (CYP7A1) and sterol 12α-hydroxylase (CYP8B1), key enzymes in classical bile acid synthesis, potentially contributing to bile acid accumulation in hepatocytes.^8,9^ Notably, temporary inhibition of CYP7A1 is physiologically required for hepatoprotection.^10^ We suggest that the sustained upregulation of miR-34a in PFIC could disrupt the physiological downregulation of CYP7A1 necessary for efficient liver regeneration; however, this association has yet to be directly confirmed in PFIC-specific models. If elevated circulating miR-34a is indeed a signature of greater disease activity in PFIC, it may serve not only as a biomarker of hepatic injury but also as a harbinger of increased cardiovascular ring to its regulatory effects on lipid metabolism and macrophage polarization (Figure 1). Future research priorities include validating miR-34a as a predictive marker in larger, genotype-characterized PFIC cohorts, defining its disease specificity relative to other cholestatic and metabolic liver disorders, and exploring targeted therapies that modulate miR-34a signalling. Looking forward, the dual roles of miR-34a in mediating both liver and vascular disease progression, through bile acid–induced toxicity, inflammation, dyslipidemia, and effects on regenerative capacity, position it as a uniquely promising candidate for multidisciplinary research at the interface of hepatology and cardiology.

**Figure 1:**
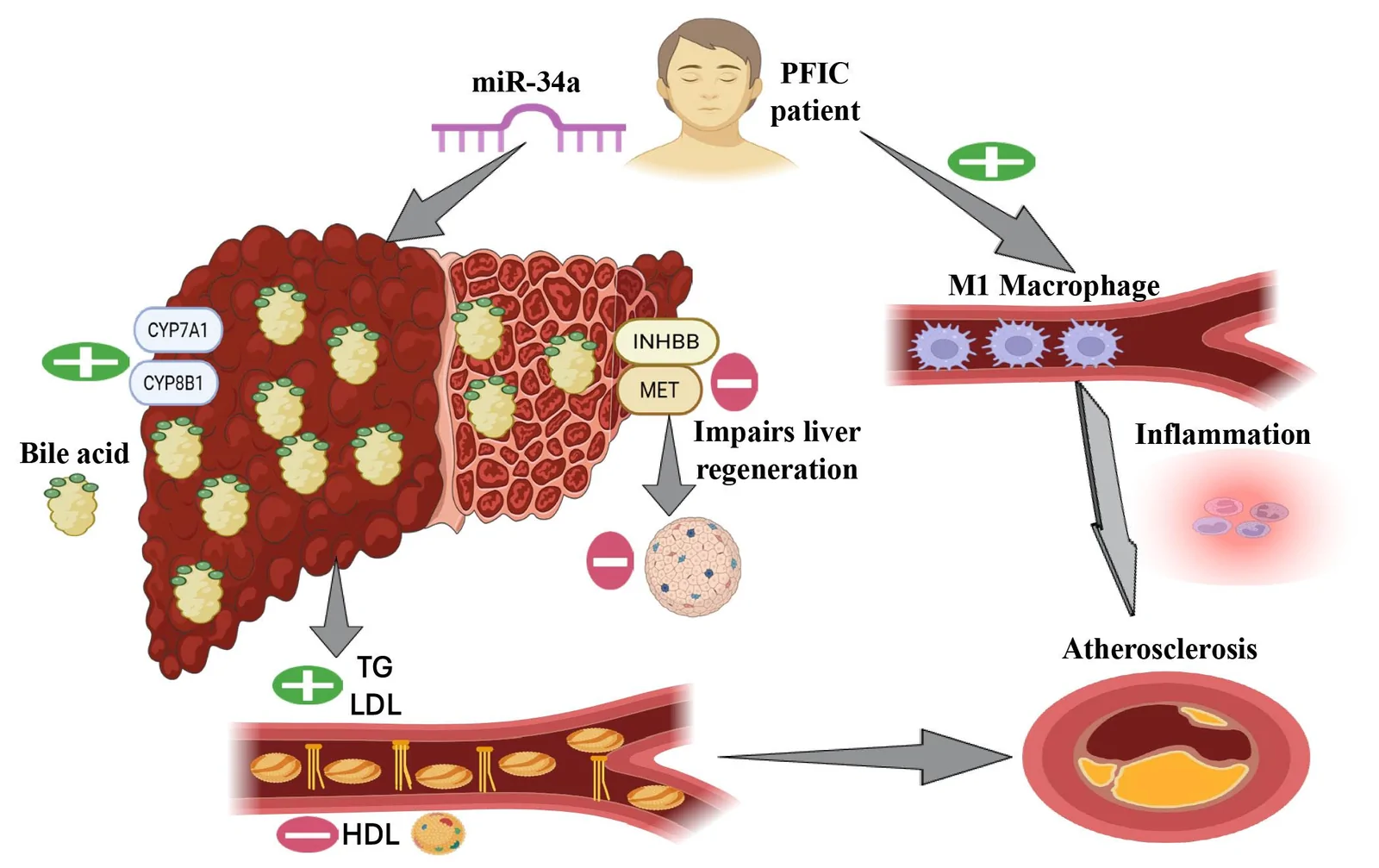
Proposed functions of miR-34a in liver and cardiovascular complications of Progressive Familial Intrahepatic Cholestasis (PFIC). Cohort studies reveal elevated serum miR-34a levels in PFIC patients. Experimental models suggest that miR-34a regulates macrophage activity and lipid metabolism, contributing to dyslipidemia and increased cardiovascular risk. miR-34a is shown to directly promote bile acid synthesis via upregulation of cholesterol 7α-hydroxylase (CYP7A1) and sterol 12α-hydroxylase (CYP8B1), while also impairing liver regenerative signaling by inhibiting hepatocyte growth factor receptor (MET) and INHBB. These pathways may underlie compromised liver adaptation and heightened cardiometabolic risk in PFIC, though direct evidence in PFIC patients remains to be established

## Competing Interests

 The authors declared no conflict of interest. All the authors read and approved the final manuscript.

## Ethical Approval

 Not applicable
